# Construction and Characterization of a Chitosan-Immobilized-Enzyme and β-Cyclodextrin-Included-Ferrocene-Based Electrochemical Biosensor for H_2_O_2_ Detection

**DOI:** 10.3390/ma10080868

**Published:** 2017-07-28

**Authors:** Wenbo Dong, Kaiyin Wang, Yu Chen, Weiping Li, Yanchun Ye, Shaohua Jin

**Affiliations:** 1 School of Material Science and Engineering, Beijing Institute of Technology, Beijing 100081, China; dwb194413@126.com (W.D.); jinshaohua@bit.edu.cn (S.J.); 2 School of Chemistry and Chemical Engineering, Beijing Institute of Technology, Beijing 100081, China; hmtan@bit.edu.cn (K.W.); ranm2017@126.com (W.L.); ye_yanchun@sina.com (Y.Y.)

**Keywords:** chitosan, catalase, cyclodextrin, ferrocene, composite electrode, electrochemical sensor

## Abstract

An electrochemical detection biosensor was prepared with the chitosan-immobilized-enzyme (CTS-CAT) and β-cyclodextrin-included-ferrocene (β-CD-FE) complex for the determination of H_2_O_2_. Ferrocene (FE) was included in β-cyclodextrin (β-CD) to increase its stability. The structure of the β-CD-FE was characterized. The inclusion amount, inclusion rate, and electrochemical properties of inclusion complexes were determined to optimize the reaction conditions for the inclusion. CTS-CAT was prepared by a step-by-step immobilization method, which overcame the disadvantages of the conventional preparation methods. The immobilization conditions were optimized to obtain the desired enzyme activity. CTS-CAT/β-CD-FE composite electrodes were prepared by compositing the CTS-CAT with the β-CD-FE complex on a glassy carbon electrode and used for the electrochemical detection of H_2_O_2_. It was found that the CTS-CAT could produce a strong reduction peak current in response to H_2_O_2_ and the β-CD-FE could amplify the current signal. The peak current exhibited a linear relationship with the H_2_O_2_ concentration in the range of 1.0 × 10^−7^–6.0 × 10^−3^ mol/L. Our work provided a novel method for the construction of electrochemical biosensors with a fast response, good stability, high sensitivity, and a wide linear response range based on the composite of chitosan and cyclodextrin.

## 1. Introduction

Reactive oxygen species (ROS) play an important role in cell signal transduction as a cell signalling molecule, and participate in the initiation of biological effects of various factors. Hydrogen peroxide (H_2_O_2_) is a representative of ROS. Its concentration is closely related to other ROS [1,2,3,4], such as superoxide anions, hydroxyl radicals, and so on. H_2_O_2_ can regulate the cell metabolism of the life system. Low concentrations of H_2_O_2_ can act as the second messenger of signal transduction and expansion, while high concentrations of H_2_O_2_ may cause damage to cell compositions and organisms, lead to oxidative stress, a variety of diseases, and physiological system disorders [5,6,7]. Therefore, H_2_O_2_ is considered as a key factor regulating the apoptosis process. Reliable and sensitive detection of H_2_O_2_ in cells is of great significance in physiology and pathophysiology [8,9]. However, the H_2_O_2_ detection at the cellular level is limited by many factors, such as small cell size, short half-life of intracellular superoxide radicals, low steady state concentrations, lack of efficient H_2_O_2_ capture probes, and so on [10].

The electrochemical techniques of enzyme sensing for H_2_O_2_ detection have attracted considerable attention due to their high sensitivity, fast response, low sample consumption, and high specificity, etc. [11]. Prasad et al. [12] developed a catalytic amperometric chip-type electrochemical biosensor device for simultaneous and real-time monitoring of the respiratory activity and H_2_O_2_ production in animal cells. Its working electrodes are comprised of platinum microelectrode and osmium-horseradish peroxidase-modified gold electrodes for the detection of oxygen consumption and H_2_O_2_ production, respectively. Inoue et al. [13] developed an electrochemical sensing device for continuous monitoring of extracellular H_2_O_2_. The device consists of an indium-tin-oxide electrode coated with osmiumpolyvinylpyridine gel polymer containing horseradish peroxidase (Os-HRP) and a poly-dimethyl siloxane well to house the cells on the chip. The extracellular H_2_O_2_ released from the cells was enzymatically reduced at the Os-HRP-modified electrode chip using Os (II) as an electron donor, resulting in a reduction of the current responses by the device. Enomoto et al. [14] described the fabrication of an electrochemical biosensor utilizing HRP-PEG-covered Au electrodes for the detection of H_2_O_2_ from cells. The miniature Au electrodes are combined with a PDMS microfluidic device to allow for the real-time measurement of ROS produced from cells. Tian et al. [15] described a biosensor which was constructed by electrodepositing HRP/PPy membrane on the surface of a ferrocene carboxylic acid mediated sol–gel–derived composite carbon electrode. Its linear range was 0.9 μM–0.2 mM, and its detection limit was 50 μM.

Chitosan is a natural polymer that has excellent biodegradability, biocompatibility, and non-toxicity [16,17,18,19,20]. Its hydroxyl and amino groups can be derivatized with many functional groups [21], which is an excellent property for the carrier of an electrochemical enzyme biosensor [22,23]. Sakuragawa et al., immobilized horseradish peroxidase (HRP) on chitosan and used it to determine trace levels of H_2_O_2_ by a luminescence method [24]. The enzyme exhibited stability and high activity for several days. Immobilization of the enzyme on chitosan provides a favourable micro-environment for the enzymatic reaction and the reactivity is well protected against acid, base, heat, and metal ion interference [25,26].

For some particular applications, electron mediators, such as ferrocene, quinone, and their derivatives, organic dyes, and so on, are doped in the chitosan membrane to improve the sensitivity of the sensor by promoting the electron transfer between the electrode and the enzyme [27,28,29]. The electron mediator with low oxidation potential distributed in the chitosan membrane can react with the reducing enzyme rapidly. The oxidation and reduction states are relatively stable, but are reversible [30,31]. Therefore, an electron channel between the enzyme activity center and the electrode can be established. Şenel immobilized HRP and ferrocene on a polymer membrane via covalent bonds and used it to modify electrode surfaces [32]. The reaction between HRP and peroxide could generate electrons that were conducted into electrode via ferrocene to amplify the current signals and improve the sensitivity of the sensor. However, electronic media are usually difficult to be dissolved in water, leading to uncontrollable doping uniformity. In addition, the doping with low uniformity can also affect the stability and service life of the sensor. The binding stability and uniformity of electronic mediators in chitosan films can be improved by chemically modifying chitosan [33]. However, the modification process is complicated and the structure of the chitosan derivatives was difficult to control.

**Figure 1:**
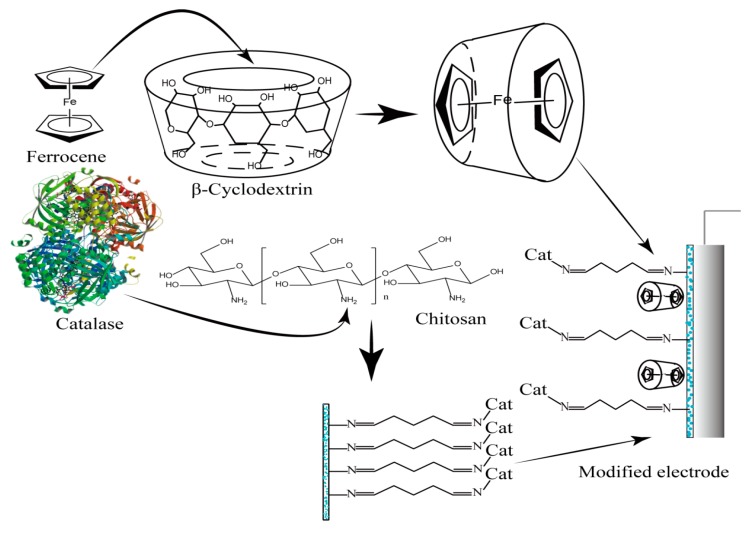
Schematic diagram of the CTS-CAT/β-CD-FE composite electrode.

Including the electron mediator inside the hydrophobic cavity of cyclodextrin, a macrocyclic oligosaccharides linked by α-1,4 glycosidic bonds with good external hydrophilicity and hydrophobicity, could promote the electron transfer from the enzyme reaction centre to the electrode surface, and avoid the loss of electron mediator and ensure sufficient mobility of mediator, thus improving the sensitivity and stability of the sensor [34,35,36]. In the present work, the electron mediator-included complex of β-cyclodextrin (β-CD-FE), was prepared to overcome the instability of the electron mediator. Additionally, catalase was immobilized on the skeleton of chitosan (CTS-CAT) by a step-by-step immobilization method to improve the immobilization efficiency and stability. Then these materials were composited on an electrode to form a CTS-CAT/β-CD-FE composite electrode that was used to detect H_2_O_2._ The above method could overcome the disadvantages of the electron-mediator-doped chitosan membrane. A novel method for the construction of electrochemical biosensors with fast response, good stability, high sensitivity, and wide linear response range based on a composite of chitosan and cyclodextrin was explored. The experimental structure diagram was shown in Figure 1.

## 2. Results and Discussion

### 2.1. Characterization of the Ferrocene-Included Complex

#### 2.1.1. Spectral Characterization of β-CD-FE

**Figure 2:**
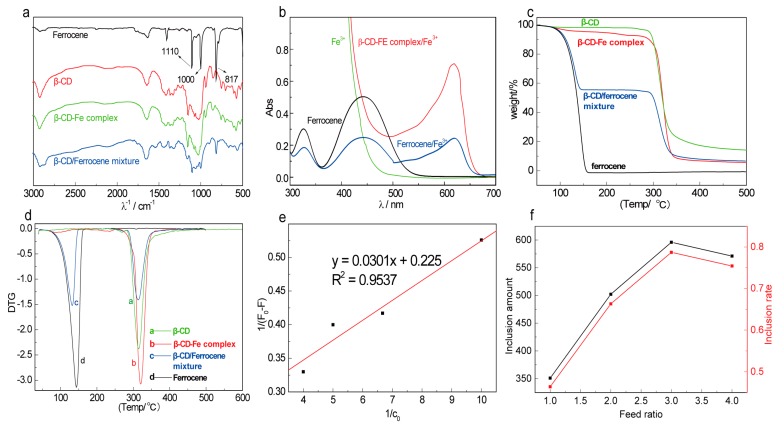
Characterization of β-CD-FE. (**a**) IR spectra of ferrocene, β-CD, the β-CD/FE mixture, and the β-CD-FE complex; (**b**) UV absorption spectra of different samples; (**c**) thermogravimetric curves of β-CD, the β-CD/FE mixture, the β-CD-FE complex, and ferrocene; (**d**) DTG curves of β-CD, the β-CD/FE mixture, the β-CD-FE complex, and ferrocene; (**e**) the standard curve for inclusion constants; and (**f**) the effects of feed ratios on the inclusion amount and the rate.

Ferrocene (FE), β-CD, the β-CD/FE mixture, and the β-CD-FE complex were respectively characterized with infrared spectroscopy and the results are shown in Figure 2a. FE exhibited three strong absorption peaks at 817 cm^−1^ (δπ_C-H_), 1000 cm^−1^ (cyclopentadienyl ring δ_C-H_), and 1100 cm^−1^ (cyclopentadienyl ring ν_C-C_) [37]. The peaks at 1156 cm^−1^ and 1028 cm^−1^ in the spectrum of β-CD were attributed to the characteristic absorption of C-O-C [38]. The characteristic absorption peaks of both FE and β-CD were observed in the mixture of FE and β-CD. However, no characteristic absorption peaks of FE were observed in the spectrum of the ferrocene-included complex formed by the solution method, indicating that FE molecules successfully entered the cavities of β-cyclodextrin.

The UV spectroscopy analysis indicated that neither the ferrocene solution nor Fe^3+^ solution could absorb visible light at 619 nm. However, ferrocene in the presence of Fe^3+^ exhibited a strong absorption peak at 619 nm. The coexistence of β-CD-FE and Fe^3+^ also resulted in a strong absorption peak at 619 nm (Figure 2b), indicating that ferrocene was included in β-CD. These results, along with the results of IR analysis, indicate that the β-CD-FE complex was successfully synthesized.

#### 2.1.2. Thermal Stability of β-CD-FE

The thermogravimetric analyses of FE, β-CD, the β-CD/FE mixture, and the β-CD-FE complex were carried out. Their TG and DTG curves are shown in Figure 2c,d, respectively. The mixture of β-CD and FE was decomposed in two steps. The weight loss of 55.3% in the temperature range of 87.7–161.9 °C was caused by the decomposition of FE and the weight loss of 38.0% in the temperature range of 288.7–348.3 °C was attributed to the decomposition of β-CD. β-CD-FE was only subjected to a rapid weight loss in the temperature range of 279.2 °C to 348 °C. The temperature at which the decomposition of β-CD-FE started was higher than the temperature (259.2 °C) required to start the decomposition of β-CD. The decomposition of β-CD-FE complex was finished at a temperature lower than that to finish the decomposition of β-CD (259.2 °C) and the weight loss of the complex was 94.0%, higher than that of β-CD (86.8%). These results indicate that the thermal stability of FE was significantly improved due to the inclusion of FE in β-CD. FE started to break down as β-CD was decomposed, which further accelerated the thermal decomposition of β-CD. These observations further confirmed that the ferrocene-included complex was successfully prepared

#### 2.1.3. Determination of Inclusion Constant, Inclusion Amount, and Inclusion Rate of β-CD-FE

As shown in Figure 2e for the standard curve of the inclusion constant, 1/(F − F_0_) is linearly related to 1/C_0_, indicating that the inclusion ratio was 1:1 [39]. The inclusion constant (K) was calculated to be 7.48 × 10^2^, suggesting that the inclusion reaction could occur spontaneously at room temperature [40].

The inclusion amounts and inclusion rates at different feeding ratios were calculated from the standard curve. The inclusion rate increased with the increase of m_FE_/m_β-CD_, peaked at the m_FE_/m_β-CD_ ratio of 3:1, and decreased as m_FE_/m_β-CD_ further increased. It can be explained that the chance for β-CD molecules contacting ferrocene molecules increased with the increase of m_FE_/m_β-CD_, resulting in more ferrocene molecules in the cavities of β-CD. Meanwhile, the increased volume fraction of ethanol in the reaction system as m_FE_/m_β-CD_ increased led to a decrease in the solubility of β-CD in the system and significantly reduced the contact between ferrocene molecules and β-CD molecules, which was unfavorable to the inclusion reaction. Therefore, β-CD-FE was prepared at m_FE_/m_β-CD_ = 3:1 in the following experiments.

Half a gram of β-CD-FE prepared at m_FE_/m_β-CD_ = 3:1 was dispersed in 20 mL of 0.1 mol/L PBS (pH = 7.0) and let stand for 3 h. Then it was precipitated and dried. It was found that the inclusion amount of FE was 98.73% to the original amount. The above results indicate that the stability of β-CD-FE in 0.1 mol/L PBS is enough to ensure the stability requirement of the composite electrode for H_2_O_2_ detection.

#### 2.1.4. Electrochemical Properties of β-CD-FE Inclusion Complex

**Figure 3:**
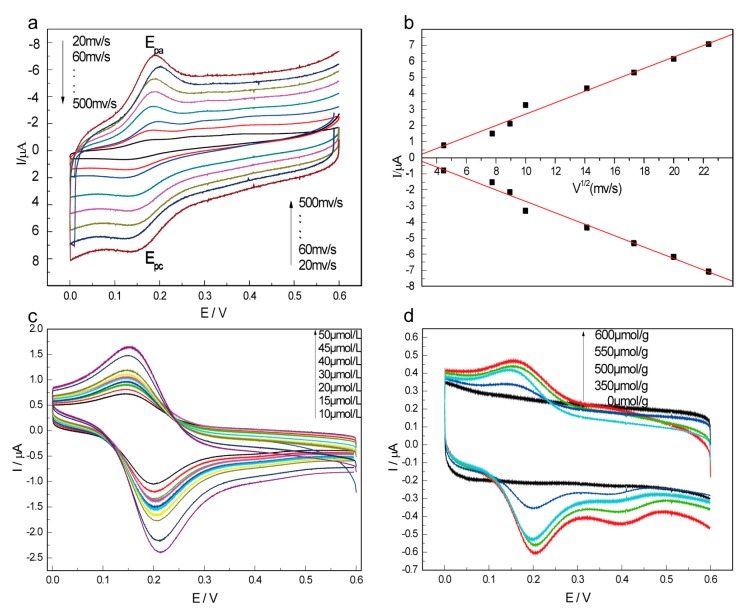
Electrochemical properties of β-CD-FE. (**a**) The cyclic voltammograms of the inclusion complexes at different sweep rates; (**b**) the relationship curve of peak current and square root of the scan rate; (**c**) cyclic voltammograms of the inclusion complexes at different concentration; and (**d**) cyclic voltammograms of the inclusion complexes at different inclusion amounts.

Figure 3a shows the cyclic voltammogram curves of β-CD-FE complex in 0.1 mol/L PBS (pH = 7.0) in the range of 0–0.60 V at different scan rates. The inclusion complex exhibited redox peaks at 0.123 V and 0.184 V, and the peak potential remained almost stable as the scan rate increased. The potential difference of the redox peaks (Δ_Ep_) is about 60 mV, close to the theoretical value of a single electron reaction, indicating that the inclusion complex possesses good redox properties.

Based on the cyclic voltammogram curves of the complex at different scan rates (Figure 3a), a linear relationship between the peak current and square root of scan rate was established, as shown in Figure 3b. These results indicate that the redox process of β-CD-FE is a diffusion process, rather than the dissociation of the FE from the cavities of the β-CD, to the surface of the electrode.

Figure 3c shows the cyclic voltammogram curves of β-CD-FE of different concentrations in 0.1 mol/L PBS (pH = 7.0) in the range of 0–0.60 V. The cyclic voltammogram curves of β-CD-FE with different inclusion amounts in 0.1 mol/L PBS (pH = 7.0) were recorded in the range of 0–0.60 V (Figure 3d). It is clear that the redox peak potential remained unchanged, but the peak current increased, with the increases of β-CD-FE concentration and the inclusion amount.

### 2.2. Optimization of Preparation Conditions for CTS-CAT

Adsorption, coating, and cross-linking are the most widely-used traditional chitosan-based enzyme immobilization methods [17]. However, the enzymes immobilized by adsorption can easily fall off due to the weak electrostatic interactions, which is not conducive to the stability and performance of the modified electrode. The carrier-binding method immobilizes an enzyme on a carrier via chemical bonding using a cross-linking agent, which forms a modified electrode with stable electrochemical properties. In general, chitosan, glutaraldehyde (GD), and enzyme are mixed together to prepare a modification solution. However, chitosan is insoluble in water and can only be dissolved in dilute acid. The cross-linking reaction is time consuming and the enzyme tends to become inactive during the reaction. In the present work, a step-by-step method was used to cross-link chitosan with GD and the cross-linked product used to immobilize CAT.

The activity of immobilized enzyme is an important factor to evaluate the performance of the chitosan biosensor. Therefore, the preparation conditions for the enzyme immobilization were optimized by monitoring the activity of CTS-CAT.

#### 2.2.1. Effects of Glutaraldehyde on Enzyme Activity

**Figure 4:**
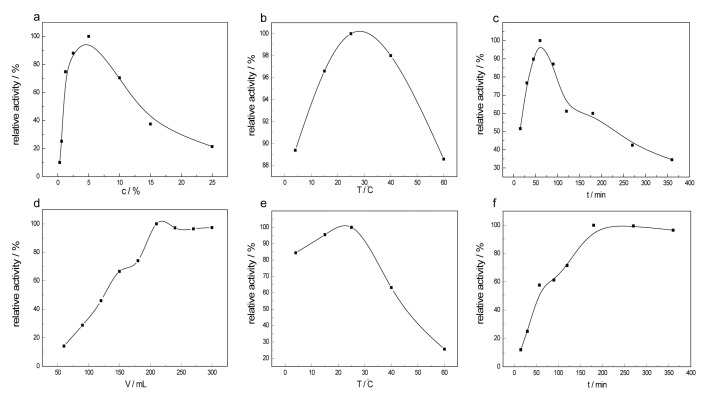
Effects of different immobilized enzyme conditions on the activity of CTS-CAT. (**a**) The effect of the cross-linking agent concentration on enzyme activity; (**b**) the effect of the modification temperature on enzyme activity; (**c**) the effect of the modification time on enzyme activity; (**d**) the effect of the enzyme amount on activity; (**e**) the effect of the immobilization temperature on enzyme activity; and (**f**) the effect of the immobilization time on enzyme activity.

Figure 4a shows the relative enzyme activity of the CTS-CAT prepared with different amounts of GD, where the highest activity is set to 100%. The activity of the immobilized enzyme increased with the increase of m_GD_/m_CTS_, peaked at m_GD_/m_CTS_ = 5%, and decreased as the m_GD_/m_CTS_ ratio further increased. For the enzyme immobilization, one of the aldehyde groups of GD reacts with the amino group of chitosan and the other one reacts with the amino group, phenol group, or the mercapto group of CAT. At low m_GD_/m_CTS_ ratios, only a limited amount of aldehyde can react with chitosan, which provided low amounts of active sites on the carrier. Only a small amount of enzyme was immobilized on the carrier. More GD reacted with chitosan as m_GD_/m_CTS_ increased, resulting in more enzyme on the carriers and, thus, higher enzyme activity. However, the high amount of cross-linked product formed at extremely high m_GD_/m_CTS_ ratios tended to undergo intramolecular or intermolecular cross-linking, which reduced the active site (aldehyde) on the carrier, increased the steric hindrance and, thus, decreased the enzyme loading. The enzyme activity was then decreased. In addition, the high amount of GD on the carrier might react with the key sites of the enzyme to form multiple bindings, which also reduced the enzyme activity. Therefore, the amount of GD was optimized as m_GD_/m_CTS_ = 5%.

#### 2.2.2. Effects of the Chitosan Modification Temperature on Enzyme Activity

The effects of the temperature for chitosan modification were determined and the results are shown in Figure 4b. The activity of catalase immobilized on chitosan increased with the increase of the modification temperature, peaked at 25 °C, and decreased as the temperature was further increased. Therefore, the temperature for the chitosan modification with GD was optimized as 25 °C, at which point the modification produced a modified chitosan that was suitable for enzyme immobilization with the highest enzyme activity.

#### 2.2.3. Effects of the Chitosan Modification Time on Enzyme Activity

As shown in Figure 4c, the activity of CTS-CAT increased with the increase of the reaction time for chitosan modification with GD, reached the highest value after a 60 min reaction, and decreased as the modification was prolonged further. It can be explained that the reactive sites for the enzyme immobilization gradually increased with the modification time, which increased the enzyme loading and, thus, the enzyme activity. As the modification time was prolonged greater than 60 min, the cross-linking degree gradually increased with the modification time and the cross-linked molecules tended to undergo intramolecular or intermolecular crosslinking. The steric hindrance was then increased which, as well as the high degree of cross-linking, led to a decrease in the adsorption efficiency of the enzyme. Therefore, the modification time of chitosan for the preparation of CTS-CAT was optimized as 60 min.

#### 2.2.4. Effects of the Enzyme Amount on Enzyme Activity

The amount of CAT for the immobilization can also affect the enzyme activity of CTS-CAT. As shown in Figure 4d, the overall enzyme activity increased with the increase of enzyme amount, reached the maximum at m_CAT_/m_CTS_ = 0.315, and remained unchanged as the enzyme amount was further increased. It can be explained that the enzyme loading increased with the increase of the enzyme amount, which increased the overall enzyme activity, until the active sites (aldehyde group) on the carrier were saturated at m_CAT_/m_CTS_ = 0.315. Further increasing the enzyme amount caused no effect on the enzyme loading or the activity of CTS-CAT. Excessive enzyme reduces the utilization efficiency of the enzyme. Therefore, the optimal amount of CAT was found to be m_CAT_/m_CTS_ = 0.315.

#### 2.2.5. Effects of the Immobilization Temperature on Enzyme Activity

The effects of immobilization temperature were determined by immobilizing CAT on the GD modified chitosan at different temperatures. The relationship between enzyme activity and immobilization temperature is shown in Figure 4e. The reaction rate for immobilization was low at low temperature, resulting in a low enzyme loading per unit time. However, low temperature is favorable to maintain the enzyme activity. Increasing the immobilization temperature can promote the thermal motion of the enzyme molecules and, thus, increase enzyme loading. However, high temperature could deactivate the enzyme and the deactivation is irreversible. Therefore, the immobilization temperature was set to 25 °C, at which point the enzyme could be completely immobilized while its activity was maintained at a reasonable value.

#### 2.2.6. Effects of the Immobilization Time on Enzyme Activity

The effects of immobilization time were investigated by immobilizing CAT on the GD-modified chitosan for different reaction times. The relationship between the enzyme activity and immobilization time is shown in Figure 4f. The extension of the immobilization time facilitates the contact and reaction between the enzyme and the aldehyde group. The activity sites (aldehydes) on the carrier were gradually saturated and the activity of the CTS-CAT became constant as the reaction time was prolonged to 180 min. The enzyme molecules on the carrier tended to block each other as the reaction time increased further, which reduced the enzyme activity. Therefore, the immobilization time of CAT on GD-modified chitosan was optimized as 180 min.

The pH of the reaction system can also affect the enzyme activity. The CTS-CAT obtained in the neutral solution exhibited the highest enzyme activity. It can be explained that free catalase has the highest activity under the conditions similar to the biological environment. Due to prolonged exposure to acidic or basic immobilization conditions may deactivate the catalase, the pH for the immobilization was set to 7 in the following experiments.

Based on these results, the preparation conditions of CTS-CAT were optimized as follows: chitosan was modified with 5% (wt %) GD at 25 °C for 60 min and the GD-modified chitosan was reacted with CAT at m_CAT_/m_CTS_ = 0.315 and pH = 7 and cured at 25 °C for 180 min.

### 2.3. Properties of the CTS-CAT/β-CD-FE Composite Electrode for H_2_O_2_ Detection

#### 2.3.1. Oxidation-Reduction Properties of CTS-CAT/β-CD-FE Composite Electrodes

**Figure 5:**
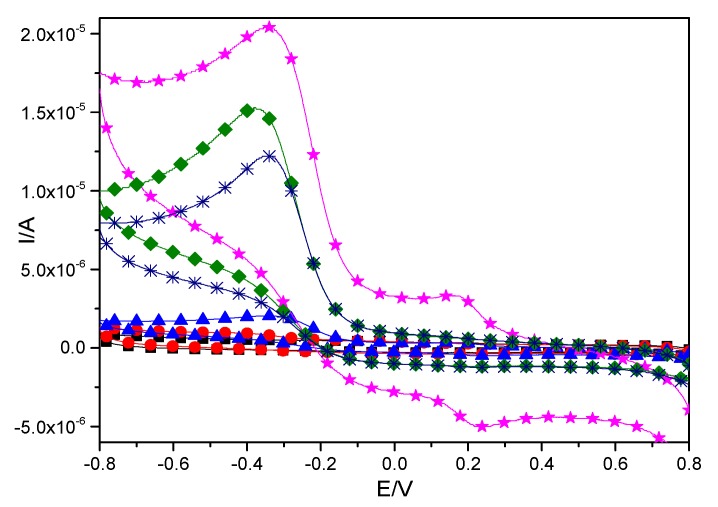
Cyclic voltammograms of the control electrodes and the CTS-CAT/β-CD-FE composite electrode in different solutions. ■: The bare electrode in 0.1 mol/L PBS (pH = 7.0) at 0.1 mol/L H_2_O_2_; ●: The control electrode B in 0.1 mol/L PBS (pH = 7.0) at 0.1 mol/L H_2_O_2_; ▲: CTS-CAT/β-CD-FE composite electrode in 0.1 mol/L PBS (PH = 7.0); ★: CTS-CAT/β-CD-FE composite electrode in 0.1 mol/L PBS (pH = 7.0) at 0.1 mol/L H_2_O_2_; ◆: The control electrode A in 0.1 mol/L PBS (pH = 7.0) in 0.1 mol/L H_2_O_2_; ✳：Control electrode A in 0.1 mol/L PBS (pH = 7.0).

Figure 5 shows the cyclic voltammetry curves of the control electrodes (bare electrode, electrode A which was coated with CTS-CAT, and electrode B coated with CTS) and the CTS-CAT/β-CD-FE composite electrode in a H_2_O_2_ solution. The procedure for preparation of different electrodes is described in Section 3.7. It could be found that neither the bare electrode without any coating nor the control electrode B coated with chitosan exhibited any obvious redox activity in 0.1 mol/L PBS (pH = 7.0) of H_2_O_2_ in the range of −0.80–0.80 V.

The control electrode A coated with CTS-CAT and the CTS-CAT/β-CD-FE composite electrode exhibited strong peak current signals in response to H_2_O_2_, indicating that they were very sensitive to H_2_O_2_. It can be explained that CAT obtained electrons from the electrode to turn into a reduced state that was oxidized back to the oxidation state by H_2_O_2_. The oxidation of CAT significantly increased the reduction peak current. The signal amplification intensity of the CTS-CAT/β-CD-FE composite electrode was significantly higher than that of electrode A, indicating that β-CD-FE played a significant role in amplifying the electrical signal and CTS-CAT/β-CD-FE electrode was more sensitive to H_2_O_2_. These results indicated that the CAT and FE included in β-CD played a major role in the response of the composite electrode to H_2_O_2_.

#### 2.3.2. Effect of pH on the CTS-CAT/β-CD-FE Composite Electrode

**Figure 6:**
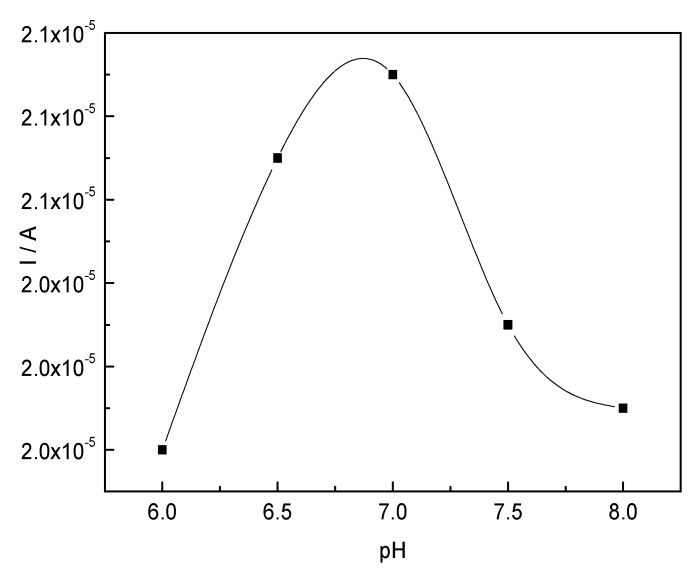
The effect of solution pH on the peak current.

Figure 6 shows the results of the peak current in the range of −0.80–0.80 V of the CTS-CAT/β-CD-FE composite electrode in the solutions of different pH conditions. The peak current increased with the increase of pH. Therefore, the optimum working pH of the CTS-CAT/β-CD-FE composite electrode was determined to be 7.0. As discussed above, the enzyme played a major role in the response of the CTS-CAT/β-CD-FE composite electrode to H_2_O_2_. Therefore, the peak current is closely related to the activity of the enzyme and the effects of working pH on peak current were similar to those of the immobilization pH on the enzyme activity.

#### 2.3.3. Sensitivity of the CTS-CAT/β-CD-FE Composite Electrodes

**Figure 7:**
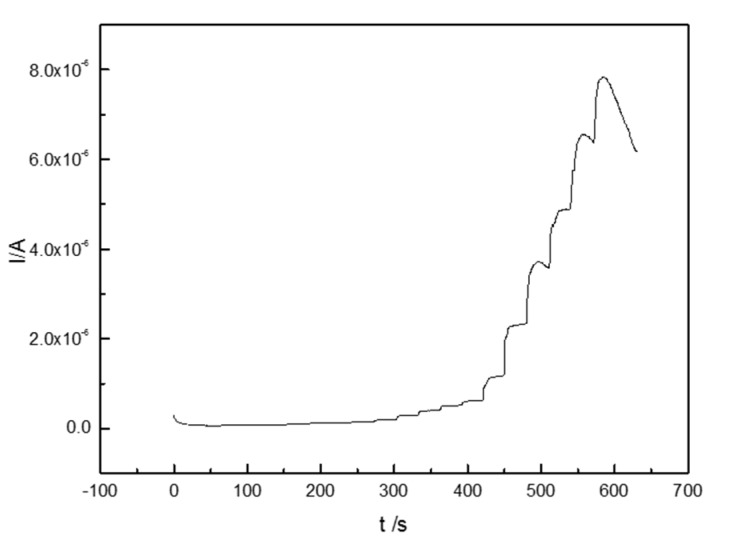
The chronoamperometric curve of the CTS-CAT/β-CD-FE composite electrode.

**Figure 8:**
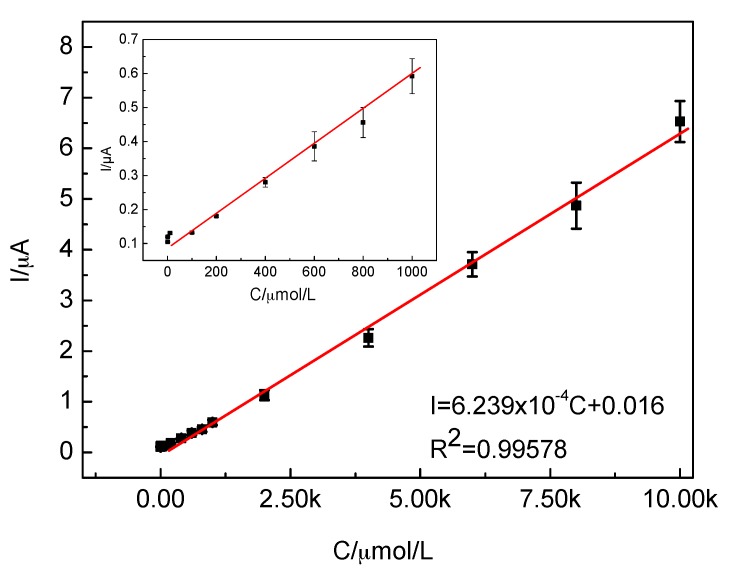
The relationship between the reduction peak current and H_2_O_2_ concentration. The reduction peak current at the low concentration is shown in the inset.

The concentrations and volumes of H_2_O_2_ used in the experiments are shown in Table 1. Figure 7 shows the time-current plot of the CTS-CAT/β-CD-FE composite electrode. The composite electrode responded to H_2_O_2_ rapidly and the reaction current reached the saturated state in less than 5 s, indicating that the activity of CAT on the composite electrode was well maintained. In addition, the peak current exhibited a linear relationship with the H_2_O_2_ concentration (Figure 8) in the range of 1.0 × 10^−7^–6.0 × 10^−3^ mol/L with the fitting equation as follows:

(1) $$I=6.239\times 10^{-4}C+0.016\;\left(R^{2}=0.99578\right)$$

where I is the peak current and C is the concentration of H_2_O_2_. Therefore, the CTS-CAT/β-CD-FE composite electrode can be used to quantitatively detect H_2_O_2_ in the range of 1.0 × 10^−7^–6.0 × 10^−3^ mol/L. The relative standard deviation of the test was 1.7–11.2% and the detection limit was 5 × 10^−^^8^ mol/L calculated at a signal-to-noise ratio of 3. The above composite electrode was stored in PBS in a fridge at 4 °C for 10 days, and then the properties of it were tested again. It was found that the decrease of the current response was 9.6%, which indicates that the stability of the composite electrode is ideal.

## 3. Materials and Methods

### 3.1. Materials

Chitosan (CTS) with a deacetylation degree of 95% and a molecular weight of 1.1 × 10^6^ was supplied by Zhejiang Aoxing Biochemical Co., Ltd. (Taizhou, China). β-cyclodextrin (β-CD) was purchased from TianJin Kwangfu Chemical Co., Ltd. (Tianjin, China). Catalase (3500 units/mg) was supplied by Shanghai Aladdin Reagent Company (Shanghai, China). Glutaraldehyde was purchased from TianJin Fuchen Chemical Co., Ltd. (Tianjin, China). Hydrogen peroxide (H_2_O_2_, 30%) was supplied by Beijing Chemical Co., Ltd. (Beijing, China). Other reagents were all analytical grade and used as received.

### 3.2. Inclusion of Ferrocene in β-CD

One gram of β-CD was added to 6.0 g of deionized water. Ferrocene was suspended in anhydrous ethanol at the mass ratio of 1:10. These two suspensions were, respectively, heated to micro-boiling, mixed at mass ratios of 1:1, 2:1, 3:1, or 4:1, stirred in an oil bath at 60 °C for 6 h, cooled to room temperature and filtered by suction filtration. The solid residue was washed with tetrahydrofuran to remove the unreacted ferrocene and dried to afford the ferrocene-included product, β-CD-FE.

### 3.3. Determination of Ferrocene Inclusion Constant, Inclusion Amount, and Inclusion Ratio

The reaction between β-CD and ferrocene can be expressed as:

(2) $$\mathrm{CD}+n\mathrm{Fe}{\left(C_{5}H_{5}\right)}_{2}={\left[\mathrm{Fe}{\left(C_{5}H_{5}\right)}_{2}\right]}_{n}-\mathrm{CD}$$

The equilibrium constant is:

(3) $$K=\left[C_{\beta -CD-FE}\right]\cdot {\left[\mathrm{CD}\right]}^{-1}\cdot {\left[\mathrm{Fe}{\left(C_{5}H_{5}\right)}_{2}\right]}^{-n}$$

The stoichiometric proportion and binding constants K, from spectrophotometric data, could be calculated using Scott’s Equation [41]:

(4) $$\frac{1}{F-F_{0}}=\frac{1}{\alpha C_{FE0}C_{\beta 0}K}+\frac{1}{{\alpha C}_{FE0}}$$

where C*_FE_*_0_ is the initial concentration of ferrocene, C*_β_*_0_ is the initial concentration of β-CD, F − F_0_ is the absorbance difference before and after β-CD is added, and *α* is the difference of molar absorptivity for free and included ferrocene.

If the resulting curve of C*_β_*_0_/F − F_0_ against C*_β_*_0_ is a straight line, the 1:1 complexing system is expected and the binding constant *K* (M^−1^) can be calculated as:

(5) K=(slope/intercept)×1000

To obtain a standard curve of UV absorption vs. ferrocene concentration, 20 mL of anhydrous ethanol, 10 mL of HCl (37 wt %), and 10 mL of Fe^3+^ solution (1.00 mg/mL) were added to a 50 mL volumetric flask. Then different amounts of ferrocene standard solutions (5.00 mg/mL) were respectively added to the volumetric flasks containing the Fe^3+^. The mixtures were allowed to stand for 30 min and their absorption at 619 nm was measured. The standard curve of UV absorption vs. ferrocene concentration can be expressed as:

(6) $$\mathrm{Abs}=1.682C_{FE.}-0.0307\;\left(R^{2}=0.9992\right)$$

To determine the inclusion amount and ratio, 0.5 g β-CD-FE was suspended in 30.00 mL anhydrous ethanol and allowed to stand for 24 h. The supernatant was collected and 20.00 mL of anhydrous ethanol was added to the β-CD-FE suspension. The suspension was allowed to stand for another 24 h and filtered. The filtrate was combined with the supernatant collected above and measured for the UV absorption at 619 nm. The ferrocene concentration was calculated from the standard curve of UV absorption vs. ferrocene concentration to determine the inclusion amount and ratio.

### 3.4. Electrochemical Properties of β-CD-FE

The cyclic voltammogram curves of β-CD-FE were measured in the potential range of 0–0.60 V using the CHI-660 electrochemical workstation of Chunghwa Instrument Co., Ltd. (Dongguan, China), by which the relationship between the electrochemical properties of β-CD-FE and FE concentration or inclusion amount were determined.

### 3.5. Preparation of CTS-CAT

CAT was immobilized on chitosan using a step-by-step method. A 2.0% chitosan solution was prepared in 1% acetic acid aqueous solution. Two milliliters of 5% glutaraldehyde (GD) solution was mixed with 1 mL chitosan solution, reacted for a certain period of time at a certain temperature, and centrifuged for 20 min at 3500 rad/min. The suspension was further filtered by suction filtration and the solid residue was washed with water to remove free GD to afford GD modified chitosan. The GD modified chitosan was added to a 3% CAT solution and reacted for a certain period of time. The suspension was then suction filtered and the unreacted CAT was removed with 0.1 mol/L PBS buffer solution to afford CTS-CAT.

### 3.6. Determination of the Enzyme Activity of CTS-CAT

The activity of CAT was determined by measuring the UV absorption spectra of a H_2_O_2_ solution at 240 nm before and after CAT was added. Enzyme activity was calculated as follows:

(7) $$P=\frac{\mathrm{AV}}{\varepsilon \mathrm{ltm}}$$

where P is the specific activity of the enzyme, A is the difference between the absorptions of the test solution and blank solution, V is the solution volume, *ε* is the extinction coefficient of H_2_O_2_ at 240 nm, l is the thickness of cuvette, t is the reaction time, and m is the mass of CTS-CAT.

### 3.7. Preparation of the CTS-CAT/β-CD-FE Composite Electrode

A glassy carbon electrode was polished to obtain a mirror surface with 0.3 μm Al_2_O_3_, sequentially followed by the ultra-sonication in deionized water, anhydrous ethanol, and deionized water for 1 min. The electrode was placed vertically and air-dried at 25 °C. A solution of 5.00 mmol/L (determined by ferrocene content) of β-CD-FE was prepared in dimethyl sulfoxide and mixed with 2 × 10^−3^ g CTS-CAT. The mixture was shaken for 30 min and 6 μL of the mixture was added dropwise to the glassy carbon electrode. The electrode was placed vertically and air-dried at 25 °C to form a modified CTS-CAT/β-CD-FE composite electrode.

Two milligrams of CTS-CAT was suspended in 2 mL of dimethyl sulfoxide to prepare control electrode A using the same procedure. Control electrode B was prepared with 6 μL of chitosan solution in acetic acid using the same procedure described above.

### 3.8. Electrochemical Properties of the CTS-CAT/β-CD-FE Composite Electrode

The cyclic voltammetry curves of different electrodes were measured at potential of 0.80–0.80 V in 0.1 mol/L PBS (pH = 7.0) and H_2_O_2_ solution, respectively, using a CHI-660 electrochemical workstation (Chunghwa Instrument Co., Ltd., Dongguan, China) to determine the electrochemical properties of control electrode A and B and the CTS-CAT/β-CD-FE composite electrode.

### 3.9. Sensitivity of the CTS-CAT/β-CD-FE Composite Electrode

The chrono-current curve of the CTS-CAT/β-CD-FE composite electrode was measured at the potential of −0.30 V in 5.00 mL 0.1 mol/L PBS (pH = 7.0) with a certain amount of H_2_O_2_ solution added at regular intervals (Table 1) using the CHI-660 electrochemical workstation. The relationship between the peak current and H_2_O_2_ concentration was established to determine the effective analysis range of H_2_O_2_ on the CTS-CAT/β-CD-FE composite electrode.

## 4. Conclusions

In the present work, ferrocene was included in the cavity of β-CD to improve its stability. The inclusion constant and thermal stability of the β-CD-FE complex were determined. The effects of the preparation conditions on the inclusion amount, inclusion rate, and electrochemical properties of the complex were evaluated. At the same time, enzyme-immobilized chitosan derivatives were prepared by a stepwise cross-linking method. The immobilization conditions were optimized by monitoring the enzyme activity as follows: Chitosan was reacted with 5% (mass fraction) GD at 25 °C for 60 min and the GD modified chitosan and CAT reacted under the condition of m_CAT_/m_CTS_ = 0.315 and pH = 7 and cured at 25 °C for 180 min. Then a glassy electrode was modified with CTS-CAT and β-CD-FE for the electrochemical detection of H_2_O_2_. The immobilized enzyme responded to H_2_O_2_ as a strong reduction peak current and β-CD-FE amplified the current signal to improve the sensitivity of the electrode. The optimal working pH for the modified electrode was 7.0. The peak current of the electrode exhibited a linear relationship with the concentration of H_2_O_2_ in the range of 1.0 × 10^−7^–6.0 × 10^−3^ mol/L. The above work constructed an electrochemical biosensor model based on enzyme-immobilized chitosan and an electron mediator-included cyclodextrin derivative to overcome the issues of a traditional chitosan-encapsulated electron mediator electrochemical sensor for the detection of H_2_O_2_.

## Figures and Tables

**Table 1:** The volume and concentration of H_2_O_2_ used in test of the sensitivity of the CTS-CAT/β-CD-FE composite electrode.

T/s	C/(mol/L)	V/(μL)	T/s	C/(mol/L)	V/(μL)	T/s	C/(mol/L)	V/(μL)
150	1.0 × 10^−4^	5	300	1.0 × 10^−1^	10	420	1.0 × 10^−1^	50
180	1.0 × 10^−3^	5	330	1.0 × 10^−1^	10	450	1.0 × 10^−1^	100
210	1.0 × 10^−2^	5	360	1.0 × 10^−1^	10	480	1.0 × 10^−1^	100
240	1.0 × 10^−1^	5	390	1.0 × 10^−1^	10	510	1.0 × 10^−1^	100
270	1.0 × 10^−1^	5	420	1.0 × 10^−1^	50	540	1.0 × 10^−1^	100
